# Patient satisfaction with private optometry service in South Africa

**DOI:** 10.4102/hsag.v31i0.3169

**Published:** 2026-06-13

**Authors:** Tinyiko P. Hlungwani, Pheagane M.W. Nkoana, Velibanti N. Sukati

**Affiliations:** 1 Department of Optometry, School of Health Care Sciences, Faculty of Health Sciences, University of Limpopo, Polokwane, South Africa

**Keywords:** satisfaction, private optometry, SERVQUAL, South Africa, variables, quality of care

## Abstract

**Background:**

Patient satisfaction plays a vital role in the healthcare sector, holding significance for both medical providers and patients.

**Aim:**

The study investigated patient satisfaction levels regarding the quality of care delivered by private optometry services in South Africa.

**Setting:**

The study was conducted across all nine provinces in South Africa, with a specific focus on individuals utilising private optometry services.

**Methods:**

This study employed a quantitative, cross-sectional design to evaluate patient satisfaction levels regarding service quality in private optometry practices within South Africa, utilising questionnaires disseminated through social media platforms for data collection.

**Results:**

Our study included 283 participants, with a mean age of 36.3 ± 10.41 years, predominantly female (69.8%) and Black African (96%), and found high satisfaction scores with optometrists. The primary reasons for visiting optometrists in our cohort were spectacle or contact lens renewal (34.9%), routine check-ups (24.5%) and addressing reduced vision (24.5%). Among those who switched optometrists, 40.8% cited relocation and 17.9% reported service dissatisfaction. The mean overall satisfaction score was 4.38 ± 0.66, with high ratings for cleanliness, physical facilities and staff conduct. Conversely, waiting time and cost received relatively lower scores.

**Conclusion:**

Participants were highly satisfied with private optometry services in South Africa, although cost and waiting time had comparatively lower ratings.

**Contribution:**

The study provides evidence on patient satisfaction in private optometry and highlights areas for service improvement.

## Introduction

Patient satisfaction is a vital concept in the healthcare industry, holding significance for both healthcare providers and patients (Ofili 2014). Kim and Park (2019) define patient satisfaction as the extend to which patients whose experiences with a healthcare organisation exceed their expectations. This metric offers valuable insights into the quality of care and patient experiences, reflecting a personal assessment of the services provided by healthcare professionals and the degree to which expectations are met and the needs are fulfilled (Bezuidenhout, Ogunsanwo & Helberg 2014; Ferreira et al. 2023). Patient satisfaction is often characterised by an emotional or practical response, where satisfaction stems from performance that surpasses expectations, and dissatisfaction arises from unmet expectations (Zhang et al. 2022). Evaluating healthcare systems requires assessing outcomes alongside patient satisfaction for a comprehensive understanding of service quality.

The healthcare landscape has undergone a notable transformation over the past two decades, with patient satisfaction emerging as a key performance indicator (Nunu & Munyewende 2017). As healthcare systems transition from traditional to fee-for-service models, patient satisfaction has become intricately linked to both financial viability and quality patient outcomes (Ferrand et al. 2016). Patient-centred care has become the gold standard, with high patient satisfaction closely tied to positive health behaviours, such as adherence to treatment plans and sustained engagement with healthcare providers (Fahmi & Raza 2013). Furthermore, satisfied patients are more likely to proactively seek medical advice and care, adhere to treatment regimes and recommend healthcare services to others (Thompson et al. 2016). In today’s competitive healthcare market, delivering high-quality, patient-centric care is crucial for healthcare providers to maintain a loyal patient base and ensure long-term success (Fahmi & Raza 2013).

Meeting patient expectations is a crucial predictor of patient satisfaction and it occurs when healthcare performance aligns with patient expectations (Batbaatar et al. 2017). In South Africa, initiatives such as the ‘BathoPele’ principles, which emphasise putting people first, establish performance standards for public sector establishments to transform their service delivery by adopting a results-driven approach, promoting innovation and shifting away from rigid, rule-based practices (Sofianos 2023). Satisfied patients are more likely to be loyal, leading to increased retention rates and potential financial benefits for healthcare practitioners. Furthermore, satisfied patients are more likely to be price insensitive and less inclined to pursue litigation. In healthcare settings where patients are satisfied, staff morale tends to be higher, leading to increased productivity and job satisfaction (Prakash 2010). The health care sector is undergoing a rapid transformation to meet the ever-increasing needs and expectations of its patient population. The level of patient satisfaction is a crucial health outcome, regarded as a key determinant of quality of care (Asamrew, Endris & Tadesse 2020).

Optometry is a regulated healthcare profession that focuses on the diagnosis, treatment and management of eye and visual conditions using various strategies, including optical devices, vision therapy and pharmaceutical agents (Naicker & Munsamy 2024; Nkoana 2024). As primary care professionals, optometrists are well positioned to address global eye care challenges because of their specialised training and comprehensive approach to vision and ocular health, enabling them to deliver accessible, high-quality eye care within communities (Okasheh-Otoom et al. 2022). The optometry profession has evolved to emphasise preventative eye care, early disease detection and therapeutic integration, with private optometry services in South Africa offering patients personalised and comprehensive care (Barrett & Loughman 2018; Black et al. 2019).

Healthcare systems, including optometry service, face challenges due to limited resources and growing demands; therefore, balancing these challenges is crucial for maintaining quality care (Faezipour & Ferreira 2013). Patient satisfaction is a key aspect of healthcare sustainability, reflecting patients’ experiences with costs, access to services and overall well-being (Faezipour & Ferreira 2013). It is used as a metric to evaluate the quality of services provided by healthcare personnel; however, the challenge lies not only in meeting patients’ requirements but also in delivering and maintaining high-quality services. Consequently, regular of patient satisfaction on healthcare systems is essential (Bezuidenhout 2015). These surveys provide healthcare providers with valuable insights into areas requiring improvement and inform policymakers about patient needs, enabling strategic planning and the delivery of effective, high-quality services (Batbaatar et al. 2017).

The Service Quality model, abbreviated SERVQUAL model, based on five dimensions – tangibles, assurance, reliability, responsiveness and empathy – has been widely used to evaluate service quality in healthcare and specifically the optometry sector (Ayikwa et al. 2023; D’cunha & Suresh 2015; Pasca & Ciavolino 2023). This framework assesses the gap between patient expectations and perceptions shaped by service encounters and organisational factors (Mason, Ngobese & Maharaj 2021). Despite the extensive literature on patient satisfaction and SERVQUAL in healthcare worldwide, there is a notable lack of research on this subject in Africa, particularly in South Africa. Previous studies have revealed resource constraints, predominantly in public-sector optometry, while the private sector, which provides services to the majority of South Africans, remains under investigated. Notably, no documented study has examined patient satisfaction with SERVQUAL model privately funded optometry services.

Hence, this study was conducted to evaluate patient satisfaction with the quality of service provided by the primary optometry service in South Africa. The SERVQUAL model was used and contextualised around the following variables: cost of service, cleanliness, complaint handling, service delivery, waiting time, product quality, information provision, staff conduct and physical facilities. The study provided valuable insights into patient experiences, informing quality improvement initiatives in healthcare settings and enhancing patient-centred care in optometry practices.

## Research methods and design

### Research design

A quantitative, cross-sectional, descriptive survey design was employed in this study. The research design serves as the plan and framework for collecting, measuring, and analysing data to address research problems (Asenahabi 2019; Ranganathan & Aggarwal 2018). It provides specific direction for connecting conceptual research problems to empirical research, determining the types of analyses required to achieve desired results (Creswell & Creswell 2014). A suitable research design was crucial for this study on patient satisfaction in private optometry service in South Africa, because it enabled the translation of the research problems into analysable data, providing answers to the questions at minimal cost while ensuring validity (Asenahabi 2019). This approach enabled a systematic investigation of patient satisfaction in South African private optometry practices.

### Study setting

Optometrists across South Africa provide private eye care services. This study involved optometry practices and their patients. Currently, around 4204 optometrists are registered with the Health Professions Council of South Africa (HPCSA) and practicing in about 2300 clinics. The number of registered private optometry clinics is approximately 3687, and this figure is steadily increasing.

### Sampling procedure

Given the considerable heterogeneity of the target population, a well-planned sampling strategy was essential for this cross-sectional study (Wang & Cheng 2020). The study used a convenience sampling approach, making the survey accessible to anyone who obtained the link.

Participants were sampled through two main channels: in-person invitations at optometry practices and online platforms, including social media (WhatsApp, Facebook and LinkedIn). To adhere to the *Protection of Personal Information Act* (POPIA) guidelines, the researcher ensured that individual optometry practices distributed the survey link without sharing client contact information. Additionally, optometrists and respondents were encouraged to share the link with others who had recently visited a private optometrist, effectively incorporating elements of snowball sampling to expand the participant pool.

### Data collection

This study used an adapted, self-administered online questionnaire based on the SERVQUAL tool (D’Cunha & Suresh 2015). The questionnaire was transferred to Google Forms (Mountain View, CA, United States [US]), a link was created and sent to collect data on participants’ demographics, their description of the consulted practice and patient satisfaction with services. It assessed five quality dimensions: reliability, tangibles, assurance, empathy and responsiveness. Responses were automatically compiled into a Microsoft Excel spreadsheet (Redmond, WA, US). The spreadsheet was imported into the IBM Statistical Package for Social Science (SPSS) software version 29 (Armonk, NY, US) for analysis. The variables measured in this study, informed by existing research (Kanan et al. 2023), are outlined in Table 1 and Table 2. This approach is consistent with similar studies, such as Turan and Bozaykut-Bük (2016), which used structured questionnaires to gather patient feedback.

**TABLE 1:** Items of the Service Quality scale questionnaire used in the study to determine patient satisfaction.

Item	Description
Tangibles (T)	T1. Services delivered (availability of equipment and examination procedure)
	T2. Physical facilities (equipment and accessibility of the facility)
	T3. Cleanliness
Responsiveness (RS)	RS4. Waiting time
	RS5. Complaint procedures
Reliability (RL)	RL1. Cost of services
	RL2. Quality of the products (spectacles and contact lenses) acquired
Assurance (A)	A1. Provision of information regarding my eye health
Empathy (E)	E1. Conduct of staff members (optometrist and front-line staff)

*Source*: Adapted from Kanan, M., Al-Khalili, R., Alshaibani, E., Saleh, Y., Assaf, R., Al-Mimi, A. et al., 2023, ‘Assessing service quality at optical centers in Palestine using SERVQUAL: Measuring ambiguity’, *Information Sciences Letters* 12(3), 1591–1607. https://doi.org/10.18576/isl/120344

**TABLE 2:** Analysis for satisfaction.

Satisfaction rate	Mean score
Very dissatisfied	1.00–1.89
Dissatisfied	1.90–2.69
Neutral	2.70–3.49
Satisfied	3.50–4.29
Very satisfied	4.30–5.00

*Source*: Adapted from Kanan, M., Al-Khalili, R., Alshaibani, E., Saleh, Y., Assaf, R., Al-Mimi, A. et al., 2023, ‘Assessing service quality at optical centers in Palestine using SERVQUAL: Measuring ambiguity’, *Information Sciences Letters* 12(3), 1591–1607. https://doi.org/10.18576/isl/120344

### Reliability and validity of the questionnaire

This study adapted and contextualised the SERVQUAL questionnaire, previously validated in studies by Al-Fraihi, Famco and Latif (2016) and Jonkisz, Karniej and Krasowska (2021), to assess service quality in healthcare. To contextualise the questionnaire for use in the current study, a panel of 10 experts, comprising optometrists, academics and private practitioners, reviewed it for validation. The Pearson product-moment correlation test confirmed the validity of each question, with values exceeding critical limits at a 0.05 significance level.

The questionnaire demonstrated high reliability, with a Cronbach’s alpha value of 0.906, indicating excellent internal consistency and suggesting that the items measured a unified construct.

The research protocol, informed by an extensive literature review and guided by Sürücü and Maslakci (2020), ensured a systematic approach. The researcher followed the protocols outlined in the research proposal, adhering to the guidelines of the University of Limpopo Turfloop Research Ethics Committee and the Postgraduate Research Manual.

### Data analysis

Data analysis combined descriptive and inferential statistical methods. Descriptive statistics, including mean scores, and standard deviations (s.d.), summarised the data’s central tendency, frequency distribution and variability. Inferential statistics, specifically *p*-values, enabled population inferences, while percentage calculations provided supplementary context for the findings.

### Ethical considerations

The study adhered to the principles of the Declaration of Helsinki and obtained ethical clearance from the Turfloop Research Ethics Committee of the University of Limpopo (ref: TREC/75/2024: PG). Participants gave consent to participate in the study by choosing ‘yes’ to agree or ‘no’ to disagree. Those who chose ‘yes’ were allowed to access the questionnaire, and those who chose ‘no’ were directed to the submit button, which redirected them to the end of the questionnaire. Participants were informed of their rights, including that participation was voluntary and that they could withdraw at any time before submitting the questionnaire.

## Results

The demographic profile of the 283 participants presented in Table 3 is characterised as follows: The mean age was 36.3 years (s.d. = 10.41), with ages spanning from 16 to 63 years. The sample predominantly consisted of females (69.8%) and Black Africans (96%). Geographically, participants were mainly from Limpopo (57.6%) and Gauteng (28.1%), with 42.1% residing in rural areas and 32.4% in urban settings. Regarding occupational status, the majority (76.6%) were employed. The participants demonstrated a high level of educational attainment, with 47.5% holding undergraduate degrees and 42.4% possessing postgraduate qualifications (Table 3).

**TABLE 3:** The socio-demographic profile of the participants.

Socio-demographic profile	Variables	Data	
		*n*	%
Gender	Female	196	69.8
	Male	84	30.2
Race	Black people	267	96.0
	Coloured people	1	0.4
	White people	3	1.1
	Indian people	7	2.6
Occupational status	Student	40	14.4
	Employed	213	76.6
	Not employed	21	7.6
	Pensioner	4	1.4
Highest education obtained	Primary	1	0.4
	Secondary	27	9.7
	Undergraduate	132	47.5
	Postgraduate	118	42.4
Type of dwelling	Rural	117	42.1
	Urban	90	32.4
	Semi-urban	71	25.5

*Source*: Adapted from Lukasiewicz, M., Gerard, S., Besnard, A., Falissard, B., Perrin, E., Sapin, H. et al., 2013, ‘Young Mania Rating Scale: How to interpret the numbers? Determination of a severity threshold and of the minimal clinically significant difference in the EMBLEM cohort’, *International Journal of Methods in Psychiatric Research* 22(1), 46–58. https://doi.org/10.1002/mpr.1379

The primary motivations for visiting an optometrist, as indicated in Table 4, were spectacle or contact lens renewal (34.9%), routine check-ups (24.5%) and addressing reduced vision (24.5%). A notable proportion of participants (13.3%) sought care for symptoms such as pain and itchiness, whereas a smaller fraction (2.9%) was referred by other healthcare professionals.

**TABLE 4:** Type of establishment, type of payment, reasons for consultation, frequency of consultations and reasons for changing optometrists.

Description	Variables	Data	
		*n*	%
Type of establishment	Solo practice	128	46.0
	Medical centre	108	38.9
	Franchise	42	15.2
Type of payment	Medical aids	117	63.7
	Cash	88	31.7
	Other	13	4.7
Reasons for consulting	Renewal of spectacles or contact lenses	97	34.9
	Reduced vision	68	24.5
	General check-up	68	24.5
	Symptoms such as pain and itchiness	37	13.3
	Referrals	8	2.9
Number of times consulting at the practice	First	98	35.2
	Second	59	21.2
	Third	31	11.2
	Multiple times	90	32.4
Reasons for changing an optometrist	Change of location	75	40.8
	Referrals	43	22.8
	Old equipment and facility layout	17	9.2
	Poor method and knowledge by an optometrist	8	4.3
	Poor treatment by the optometrist and staff	3	1.6
	The practitioner or receptionist is rude	6	3.3
	Less impressed with spectacles or contact lenses received	33	17.9

Table 4 presents findings on type of establishment, type of payment, reasons for consultation, frequency of consultations and reasons for changing optometrists. Regarding changes in optometrists, the key factors driving this decision included relocation (40.8%) and recommendations from others (22.8%). Dissatisfaction with services, encompassing issues, such as subpar spectacles or contact lenses (17.9%), outdated equipment and facilities (9.2%), perceived knowledge gaps among optometrists (4.3%) and poor staff treatment (1.6%), also contributed to the decision to switch. Furthermore, 3.3% of participants cited unprofessional behaviour from practitioners or receptionists as a reason for changing optometrists.

The results further indicate that 35.2% of patients visited the practice for the first time, while 21.2% and 11.2% had their second and third consultations, respectively. Notably, 32.4% of patients had multiple consultations, suggesting a significant proportion of repeat patients.

The distribution of consultations across different types of establishments reveals that solo practices accounted for 46% of consultations, followed by medical centres at 38.9% and franchises at 15.2%. This suggests that solo practices are the most common setting for consultations in this sample. An examination of payment methods reveals that medical aids were the primary payment method, accounting for 63.7%. Cash payments accounted for 31.7% of all consultations, while other payment methods comprised 4.7%.

Table 4 outlines the motivations behind participants’ decisions to visit an optometry practice and the factors that led them to switch to another practice (adapted from D’Cunha & Suresh 2015).

The study’s findings, as presented in Table 5, indicate an overall satisfaction mean score of 4.38 ± 0.66. A breakdown of patient satisfaction across various domains revealed the following mean scores 4.60 ± 0.69 for cleanliness, 4.52 ± 0.78 for physical facility, 4.41 ± 0.90 for product quality, 4.36 ± 0.87 for complaint handling, 4.50 ± 0.74 for staff conduct, 4.41 ± 0.85 for service delivery, 4.35 ± 0.95 for information provision, 4.24 ± 0.97 for waiting time and 4.05 ± 1.07 for cost of service.

**TABLE 5:** The descriptive statistics of patient satisfaction levels.

Variables	Mean	s.d.	Percentage
Cost	4.05	1.07	81.0
Waiting time	4.24	0.97	84.4
Service delivered	4.41	0.85	88.2
Provision of information	4.35	0.95	87.0
Conduct of staff members	4.50	0.74	90.0
Handling of complaints	4.36	0.87	87.2
Quality of product	4.41	0.90	88.2
Physical facility	4.51	0.78	90.2
Cleanliness	4.60	0.69	92.0
Overall satisfaction	4.38	0.66	87.6

*Source*: Adapted from Alibrandi, A., Gitto, L., Limosani, M. & Mustica, P.F., 2023, ‘Patient satisfaction and quality of hospital care’, *Evaluation and Program Planning* 97, 102251. https://doi.org/10.1016/j.evalprogplan.2023.102251

s.d., standard deviation.

As shown in Table 6a and Table 6b, satisfaction was affected by age (*r* = 0.172; *p* = 0.004), race (*r* = 0.118; *p* = 0.049), occupational status (*r* = 0.108; *p* = 0.072 [significant at *p* = 0.1]), the number of times the patient consulted at the facility (*r* = 0.161; *p* = 0.007), whether they consulted elsewhere before (*r* = 0.107; *p* = 0.075 [significant at *p* = 0.1]) and whether they had changed optometrists (*r* = 0.209; *p* = 0.004).

**TABLE 6a:** Correlation between demographic information and satisfaction.

Variable	*r*	*p*-value
Age	0.172	0.004
Gender	−0.041	0.497
Race	0.118	0.049
Province of origin	−0.078	0.197
Type of dwelling	0.001	0.982
Highest educational level	−0.066	0.274
Occupational status	0.108	0.072*
Type of establishment	−0.060	0.316
Frequency of consultations at the facility	0.161	0.007
Previous consultations at another facility	0.107	0.075*
Change of optometrist or facility	0.209	0.004
Reason for this consultation	0.065	0.280
Type of payment	−0.0040	0.504

Note:

* , Significance at *p* = 0.10.

**TABLE 6b:** Correlation between demographic information and satisfaction.

Variable	*t*-statistic	*p*-value
Gender	−0.680	0.496

## Discussion

The study aimed to determine the level of patient satisfaction with the quality of care delivered by primary optometry services in South Africa. It applied the SERVQUAL model and adapted it to include the cost of service, cleanliness, complaint management, service delivery, waiting time, product quality, information provision, staff behaviour and the condition of physical facilities. The study supports evidence-informed decisions that can improve patient outcomes, increase service efficiency and promote a more patient-centred approach to eye care (Batbaatar et al. 2017).

The demographic characteristics of the sample show a predominance of female participants, accounting for 69.8% of the total. This finding aligns with previous research indicating that females tend to have higher response rates in online surveys (Wu, Zhao & Fils-Aime 2022). Similar studies have reported a higher proportion of female participants, such as Larose and Tsai (2014), who found that 60.7% of their 136 participants were female, and Malińska and Bugajska (2021), who observed a greater number of female participants across various age groups.

The mean age of the participants was 36.3 ± 10.41 years, aligning with the working-age population and mirroring the target population in various studies (Lorenti et al. 2020). Tessema and Adane’s (2015) study reported a comparable mean age of 34.9 years, further supporting the validity of our sample’s demographics. The employment rate among participants was high, at 76.6%, consistent with Statistics South Africa’s (Stats SA 2024) findings. This is notable, given that employed individuals are more likely to participate in surveys compared to the broader population (Saleh & Bista 2017).

**FIGURE 1:**
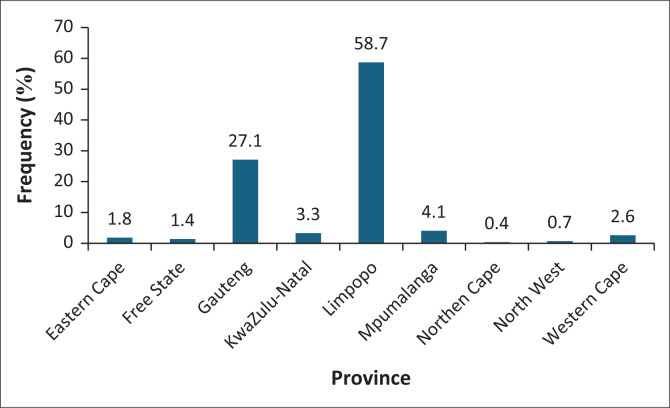
Distribution by province (*n* = 278).

The sample’s geographical distribution is also noteworthy (Figure 1). The sample largely represented people from Limpopo province, with notable representation from Gauteng as well, reflecting the population sizes of these provinces (Stats SA, Census 2022). The demographic breakdown of the sample indicates 42.1% of participants resided in rural areas, aligning with South Africa’s context, where rural populations are significant (Mubangizi 2023). However, this contrasts with studies such as Ibanga et al. (2017), in which urban respondents predominated.

The study’s findings indicated high satisfaction levels with various aspects of healthcare services. The overall satisfaction score was 4.38 ± 0.66, suggesting that patients were very satisfied and reflecting a predominantly positive experience. This aligns with existing literature reporting high satisfaction levels in healthcare settings (Batbaatar et al. 2017; Berkowitz 2016). This may be attributed to the findings of high SERVQUAL reported in the related study (Hlungwani, Nkoana & Sukati 2025).

Specifically, participants expressed high satisfaction with the cleanliness of the facilities (mean score of 4.60 ± 0.69), the layout of the physical facilities (mean score of 4.51 ± 0.78) and the conduct of healthcare providers (mean score of 4.50 ± 0.74). These findings are consistent with prior research highlighting the importance of these factors in shaping patient satisfaction (Ibanga et al. 2017; Chowdhury et al. 2024). Service quality reviews routinely identify cleanliness, facility layout and personal interaction as key elements shaping patients’ perceptions (Batbaatar et al. 2017). Previous studies suggest that a clean and organised healthcare setting enhances patient comfort and trust (Al-Abri & Al-Balushi 2014; Donabedian 2005). It also highlights that respectful communication and professionalism are among the most influential factors in patient satisfaction (Agarwal & Babu 2025; Kanwel et al. 2024). The findings of this study on staff conduct are consistent with existing research, which reported 95.80% of patients were highly satisfied with courtesy and respect, and 89.92% with practitioners’ attentiveness (Tessema & Adane 2015). However, another study suggests that the quality of treatment provided by non-physician staff requires enhancement (William 2021).

Patients’ satisfaction with service delivery (mean score of 4.41 ± 0.85) indicated overall satisfaction with efficiency, consultation flow and care organisation. Prior studies found that a well-coordinated service procedures enhance patient satisfaction and reduce the perceived burden of seeking care (Bleich, Özaltin & Murray 2009; Manary et al. 2013).

The mean score on provision of information was 4.35 ± 0.95, suggesting that patients felt sufficiently informed about their conditions, available treatments and follow-up care. Additionally, research shows that in primary health settings, effective and culturally sensitive communication increases patient comprehension and satisfaction (Ibrahim, Sidani & Garcia 2020).

Patients’ confidence in the fairness and responsiveness of the service was indicated by a mean complaint-handling score of 4.36 ± 0.87. Effective handling of patient complaints is essential to patient safety, especially in areas requiring aftercare, such as contact lenses, and for ongoing development (Pichert, Hickson & Moore 2013; Reader, Gillespie & Roberts 2014). When the complaint-handling processes are transparent, they improve patient confidence in medical services (Hsieh 2010).

The high level of satisfaction with product quality (mean score of 4.41 ± 0.90) indicated that consumers have a favourable opinion of the optical products and spectacles they received. Product quality is a known factor in determining patient satisfaction in optometry because it affects comfort, vision and continued use of optical devices (Goi, Ahmad & Goh 2021; Liu & Mallonee 2019). According to studies conducted in low- and middle-income settings (Ramke et al. 2017), reliable, long-lasting optical products boost patient confidence in public sector eye care services.

The study revealed that patients were satisfied with the waiting times (4.24 ± 0.97) and the cost of the service (4.05 ± 1.07). While both areas were rated favourably, these scores may still be improved. According to studies by Bleustein et al. (2014) and Bleich et al. (2009), longer waiting times and ineffective service can lower patient satisfaction even when clinical care is thought to be good. A common observation among optometrists running multiple facilities and moving between them based on where patients are located is that this may result in longer transit times, thereby increasing waiting times. Careful planning of appointments and patient flow has been found to shorten wait times and boost satisfaction (Huang, Yong & Wu 2019; Thompson et al. 2012). The cost of services is a significant component of patient satisfaction, particularly in eye care, where access and treatment continuation may be impacted by affordability. Although patients in this study considered the cost as acceptable, the lower mean score compared with other factors may reflect the financial strain many patients face. Small out-of-pocket expenses whether as full payments or split payments for medical aid shortfalls, can have an impact on follow-up care and the uptake of optical devices and medication in low- and middle-income settings (Palagyi et al. 2016; Ramke et al. 2017). Providers may use effective methods that enhance perceptions of affordability, including targeted financial support for vulnerable groups, have consistent cost structures, and also clear pricing communication (Amin, Chong & Manaf 2020). Improving these aspects could boost patient trust and encourage ongoing use of optometry services.

The findings in Table 6a and Table 6b show that several demographic and service use factors influenced patient satisfaction. Age showed a significant association with satisfaction (*r* = 0.172; *p* = 0.004), indicating that older patients tended to report higher satisfaction levels. Similar trends have been documented in broader healthcare research, where older adults often express more positive evaluations of care due to differing expectations, more frequent interactions with health services, and greater appreciation of providers’ effort (Bleich et al. 2009; Manary et al. 2013).

Race also demonstrated a significant though modest association with satisfaction (*r* = 0.118; *p* = 0.049). Differences in cultural background, communication needs and prior encounters with the health system may shape how patients perceive the quality of care and interpersonal interactions. Studies have shown that cultural responsiveness and equitable treatment influence perceived fairness and satisfaction (Doyle, Lennox & Bell 2013; Ibrahim et al. 2020).

The association between occupational status and satisfaction (*r* = 0.108; *p* = 0.072) did not reach the conventional significance threshold but suggests a possible trend. Social and economic factors often influence expectations, access to resources and attitudes towards health services, which can shape satisfaction outcomes. Previous work highlights that socioeconomic status can influence how patients judge the value and accessibility of care (Amin et al. 2020; Berkowitz 2016).

Satisfaction was affected by repeat consultations (*r* = 0.161; *p* = 0.007). As patients consult repetitively, they are more likely to develop trust and comfort with the practitioner or facility. Their familiarity with the provider and the process may be beneficial, increasing the likelihood of assurance. This observation is common to other studies in which continuity of care was assumed to enhance communication, trust and patient adherence (King & Hoppe 2013; Street et al. 2009).

Previous consultation elsewhere showed a marginal association with satisfaction (*r* = 0.107; *p* = 0.075). Patients tend to base their expectations on previous experiences, comparing them with those expectations. Satisfaction rating may rely more on the normative reference from the previous exposures, hence with more attention to detail on aspects such as resources. Numerous studies suggest that observations on prior experiences affecting current satisfaction may be based on expectation framing and on how quality is perceived (Al-Abri & Al-Balushi 2014; Doyle et al. 2013).

Satisfaction was affected by whether patients consulted elsewhere before the current consultation (*r* = 0.209; *p* = 0.004). The needs of the patients may have been met when they consulted with one practitioner and then another. Service from the new practitioner could have been deemed better or more aligned with the needs of the patient. Hence, this supports the belief that trust, communication and stability, stemming from assurance of the new provider contribute strongly to overall satisfaction (Batbaatar et al. 2017; Reader et al. 2014).

### Recommendations

This study has focused on patient satisfaction based on elements of SERVQUAL. Future studies should also explore the impact of unacceptable behaviour, such as poor treatment by staff and rudeness from practitioners or receptionists, on patient satisfaction and loyalty. Furthermore, the role of consistency in upholding high standards of patient care and its impact on competitiveness and profitability should be examined (Bhati, Deogade & Kanyal 2023).

Additionally, research should investigate the implementation of innovative business models, transparent billing practices, financial counselling and payment plans to enhance patient satisfaction (Erickson et al. 2020; Whaley et al. 2014). By understanding these factors, optometrists can tailor their services to meet patient needs and expectations, ultimately enhancing satisfaction and loyalty.

### Limitations of the study

This investigation acknowledges certain limitations, primarily stemming from its sample size, which might not accurately reflect the broader demographic. The study’s reliance on self-reported data could also influence the outcomes. The sample’s demographic breakdown, including geographical location and racial distribution, may also limit the generalisability of the findings to other contexts. To validate the findings, future studies should employ larger, more diverse samples and mixed-methods data collection approaches across various settings. Given the potential impact of geographic location on healthcare satisfaction, additional research is warranted to include a broader range of demographics and locations.

## Conclusion

It can be concluded that demographic characteristics and prior eye care experiences influence patient satisfaction with optometry services. While occupational status showed a weaker relationship with satisfaction, age and race had significant associations. Additionally, patterns show that patients who were more frequent users of the facility or who had previously interacted with different providers based their level of satisfaction on these prior experiences. These patterns aligned with the body of research showing that satisfaction is influenced by both expectations and comparisons to prior care.

Patients were generally satisfied, particularly with waiting times and cost, which could be improved. Strengthening service consistency, improving patient communication and addressing the specific expectations of different demographic groups may further enhance patient satisfaction. Continuous monitoring of patient experiences remains important to maintain quality care and to ensure responsive and patient-centred optometry services.
